# Annotation of nuclear lncRNAs based on chromatin interactions

**DOI:** 10.1371/journal.pone.0295971

**Published:** 2024-05-06

**Authors:** Saumya Agrawal, Andrey Buyan, Jessica Severin, Masaru Koido, Tanvir Alam, Imad Abugessaisa, Howard Y. Chang, Josée Dostie, Masayoshi Itoh, Juha Kere, Naoto Kondo, Yunjing Li, Vsevolod J. Makeev, Mickaël Mendez, Yasushi Okazaki, Jordan A. Ramilowski, Andrey I. Sigorskikh, Lisa J. Strug, Ken Yagi, Kayoko Yasuzawa, Chi Wai Yip, Chung Chau Hon, Michael M. Hoffman, Chikashi Terao, Ivan V. Kulakovskiy, Takeya Kasukawa, Jay W. Shin, Piero Carninci, Michiel J. L. de Hoon

**Affiliations:** 1 RIKEN Center for Integrative Medical Sciences, Yokohama, Japan; 2 Autosome.org, Russia; 3 FANTOM Consortium, Dolgoprudny, Russia; 4 Institute of Medical Science, The University of Tokyo, Tokyo, Japan; 5 College of Science and Engineering, Hamad Bin Khalifa University, Doha, Qatar; 6 Center for Personal Dynamic Regulome, Stanford University, Stanford, California, United States of America; 7 Department of Biochemistry, Rosalind and Morris Goodman Cancer Research Center, McGill University, Montréal, Québec, Canada; 8 RIKEN Preventive Medicine and Diagnosis Innovation Program, Wako, Japan; 9 Department of Biosciences and Nutrition, Karolinska Institutet, Huddinge, Sweden; 10 Stem Cells and Metabolism Research Program, University of Helsinki and Folkhälsan Research Center, Helsinki, Finland; 11 RIKEN Center for Life Science Technologies, Yokohama, Japan; 12 Division of Biostatistics, Dalla Lana School of Public Health, University of Toronto, Toronto, Ontario, Canada; 13 Department of Computer Science, University of Toronto, Toronto, Ontario, Canada; 14 Advanced Medical Research Center, Yokohama City University, Yokohama, Japan; 15 Department of Statistical Sciences, University of Toronto, Ontario, Canada; 16 The Centre for Applied Genomics and Program in Genetics and Genome Biology, The Hospital for Sick Children, Toronto, Ontario, Canada; 17 Princess Margaret Cancer Centre, Toronto, Ontario, Canada; 18 Department of Medical Biophysics, University of Toronto, Toronto, Ontario, Canada; 19 Vector Institute, Toronto, Ontario, Canada; 20 Genome Institute of Singapore (GIS), Agency for Science, Technology and Research (A*STAR), Singapore, Republic of Singapore; 21 Human Technopole, Milan, Italy; Teikyo University, School of Medicine, JAPAN

## Abstract

The human genome is pervasively transcribed and produces a wide variety of long non-coding RNAs (lncRNAs), constituting the majority of transcripts across human cell types. Some specific nuclear lncRNAs have been shown to be important regulatory components acting locally. As RNA-chromatin interaction and Hi-C chromatin conformation data showed that chromatin interactions of nuclear lncRNAs are determined by the local chromatin 3D conformation, we used Hi-C data to identify potential target genes of lncRNAs. RNA-protein interaction data suggested that nuclear lncRNAs act as scaffolds to recruit regulatory proteins to target promoters and enhancers. Nuclear lncRNAs may therefore play a role in directing regulatory factors to locations spatially close to the lncRNA gene. We provide the analysis results through an interactive visualization web portal at https://fantom.gsc.riken.jp/zenbu/reports/#F6_3D_lncRNA.

## Introduction

Human cells express tens of thousands of long non-coding RNAs (lncRNAs) [1], defined as RNA transcripts of at least 200 nt with no or limited protein-coding potential. Although this class of RNAs has been known for almost 50 years [2–4], ~95% of lncRNAs lack functional annotations or detailed characterization to establish whether they have any biological role [5], though some lncRNAs have been shown to have important roles in transcriptional regulation [6,7], chromatin maintenance [8,9], translation [10], and other biological processes. Exploring the role of lncRNAs systematically is challenging due to their low expression [11], rapid degradation compared to mRNAs [12,13], high cell type-specificity [11], and lack of conservation across organisms [14]. In contrast to protein-coding genes, the absence of families of lncRNAs with related sequences further hinders their classification.

Even in low copy numbers, lncRNAs can initiate the formation of nuclear compartments by forming scaffolds to interact with RNA binding proteins (RBPs) and other mediator proteins, and can regulate transcription and chromatin remodeling [15–22]. Previously, reporter assays and qPCR quantification after siRNA knockdown of lncRNAs showed differential expression of protein coding genes located within a 300 kb genomic region of the lncRNA gene [23]. Studies using microscopy and RNA-chromatin interaction sequencing data have identified several nuclear lncRNAs constrained to regions close to their gene of origin in three-dimensional space [24,25]. These target regions can be several megabases away in linear genomic distance [26–30] but nearby in physical space, as distal genomic regions are brought into spatial proximity by chromatin folding.

We created high-resolution genomic interaction maps using newly generated Hi-C data for induced pluripotent stem cells (iPSCs), as well as previously published Hi-C data from 17 other human cell types and tissues. By integrating Hi-C data with RBP interaction data, we show that lncRNAs may guide RBPs to the promoters of the lncRNA target genes.

A visualization platform is provided on ZENBU-Reports that allows users to browse and compare the biological features and predicted interactions of each lncRNA in individual cell types (https://fantom.gsc.riken.jp/zenbu/reports/#F6_3D_lncRNA).

## Results

### Defining candidate target genes for nuclear lncRNAs

To identify nuclear lncRNAs in iPS cells, we first estimated the relative prevalence of each lncRNA in the nucleus using subcellular RNA sequencing data generated from RNA isolated from each subcompartment. In total, 3,474 out of 4,460 lncRNAs expressed in iPSc were enriched in the nucleus, with a nuclear-to-cytoplasmic expression ratio ≥ 0.5 (see Methods; **S1 Table**). Next, to identify the genomic distance over which nuclear lncRNAs bind, we used previously published deep sequenced data of RADICL-seq [31], an RNA-chromatin proximity assay, in iPSC [32]. To remove nascent RNAs, paired reads with either the RNA tag mapping to an intron or the DNA tag overlapping with the gene of origin were discarded (**S1 Fig**). The statistical significance of a lncRNA forming an RNA-chromatin interaction at a specific genomic location was determined using a binomial test (**FDR ≤ 0.01**; see Methods), with a background probability calculated from inter-chromosomal binding events [33] (see Methods for details).

**Fig 1:**
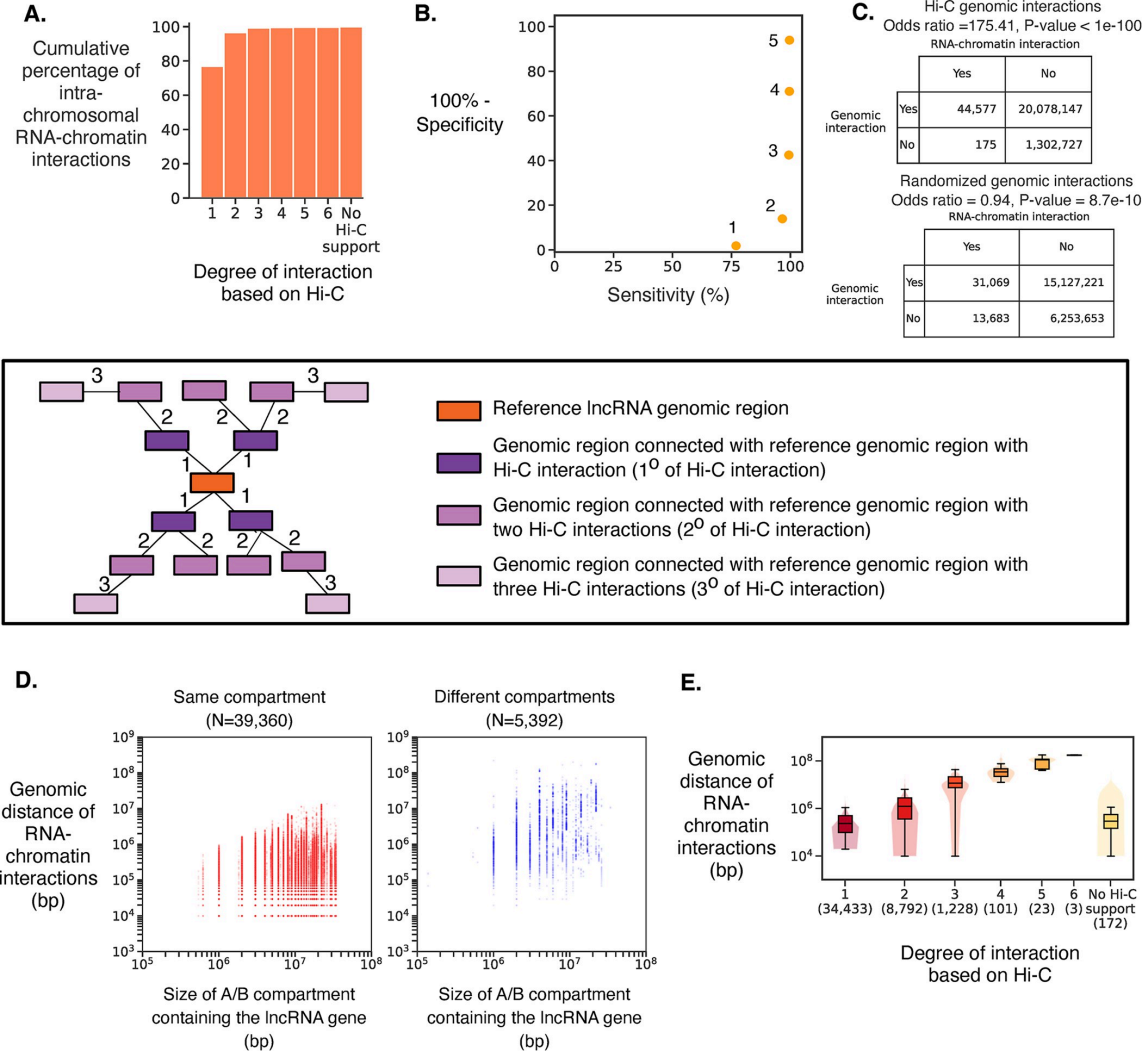
Comparison between nuclear lncRNA Hi-C genomic interaction and intra-chromosomal RNA-chromatin interactions in iPSC. **(A)** Cumulative distribution plot showing the degree of Hi-C interactions between the nuclear lncRNA gene and chromatin regions where the lncRNA binds. **(B)** The relationships among sensitivity, specificity, and degree of Hi-C interaction to identify the RNA-chromatin interaction using Hi-C genomic interactions. The x-axis represents sensitivity and y-axis represents 100%-specificity to identify RNA-chromatin interactions using Hi-C interactions. The degree of Hi-C interaction is shown next to each dot. **(C)** Contingency table showing enrichment of RNA-chromatin interactions in genomic regions supported by Hi-C interactions (top panel) and random interactions (lower panel) for the two degrees of Hi-C interactions calculated using the two-sided Fisher’s exact test. (**D)** Linear genomic distance between the lncRNA gene and its RNA-chromatin interactions in the same and different A/B. The x-axis represents the A/B compartment size, and the y-axis represents the genomic distance between the lncRNA gene and RNA-chromatin interaction. Each dot is one RNA-chromatin interaction. (**E)** Linear genomic distance between lncRNA gene and its RNA-chromatin interaction for the individual degree of separation based on Hi-C interactions.

**Fig 2:**
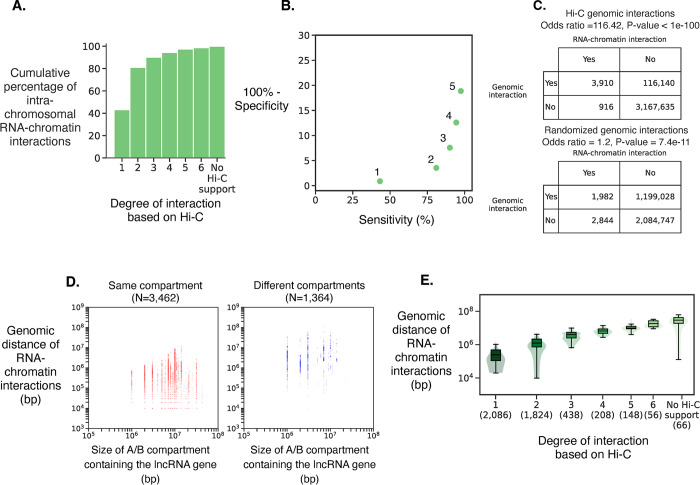
Comparison between nuclear lncRNA Hi-C genomic interaction and intra-chromosomal RNA-chromatin interactions in K562. See the caption of **Fig 1** for a description of these panels.

In iPSC, 1,970 lncRNAs have significant intra-chromosomal RNA-chromatin interactions; 1,723 are nuclear lncRNAs (**S1A Fig**). 97.15% of the genomic regions with which nuclear lncRNAs interact are within two degrees of Hi-C interactions from the gene encoding the lncRNA (**Fig 1A**). Based on the specificity-sensitivity curve we selected genomic regions that are up to two degrees of Hi-C interactions (**Fig 1B**). Further, RNA-chromatin interactions show a significant enrichment in Hi-C interactions, while enrichment is lost if the genomic interactions are randomized (**Fig 1C**). This demonstrates that spatial proximity of the genomic regions to a lncRNA gene plays an important role in determining its target region. While 88.56% of RNA-chromatin interactions are in the same A/B compartment as the lncRNA (**Fig 1D**), RNA-chromatin interactions may occur over several megabases in linear distance, indicating that chromatin-interacting lncRNAs are typically within their 3D spatial neighborhood but are not necessarily restricted to their immediate genomic neighborhood (**Fig 1E**). The significant RNA-chromatin interactions in three other human cell types (cell lines K562, MDA231 and MM1S), for which previously published RNA-chromatin interaction data were available [33,34] (**S1B and S1C Fig),** also show that the majority of lncRNAs bind to chromatin locally. In K562 Hi-C interactions similarly showed that the genomic regions with which a nuclear lncRNA interacts are spatially close to the lncRNA gene (**Fig 2A–2E)**. We performed GWAS SNP enrichment analysis in each A/B compartment to provide an annotation of the potential phenotypic relevance of lncRNA genes in the compartment (FDR ≤ 0.1; **S2 Table**).

**Fig 3:**
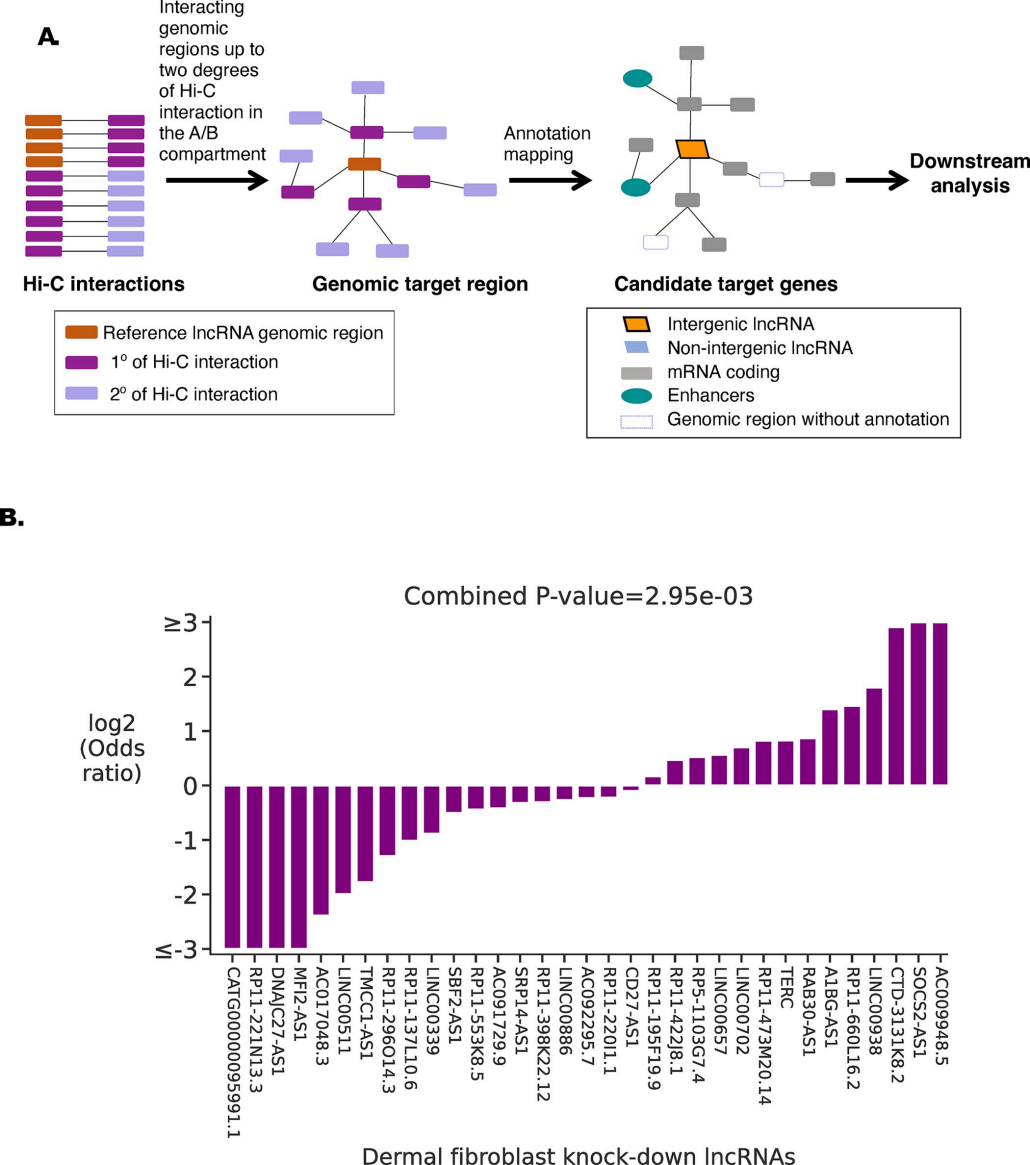
Nuclear-lncRNAs candidate target genes. **(A)** Schematic diagram showing the workflow to identify the candidate target genes of a lncRNA using Hi-C. (**B)** Enrichment of upregulated or downregulated genes in the target genes upon lncRNA knock-down in dermal fibroblasts calculated using the two-sided Fisher exact test. The y-axis represents log2 (Odds ratio). A positive value represents enrichment (Odds ratio >1) of upregulated genes, and in contrast, a negative value (Odds ratio <1) represents the enrichment of downregulated genes in candidate target genes due to the lncRNA knock-down (x-axis).

Next, we calculated Hi-C interactions for 16 other cell types for which deep sequenced Hi-C data were available (one embryonic cell line, six primary cell types, eight cell lines, and one tissue type**; S3 Table)**. Based on Hi-C interaction data, for all nuclear lncRNAs, we selected genomic regions within two degrees of Hi-C interactions from the lncRNA gene as candidate target regions (**Fig 3A**). To assess if candidate target genes located in these regions may be regulated by the lncRNA, we used previously published CAGE transcriptome sequencing data generated after knocking down lncRNAs using antisense oligos [35]. Upon knockdown, 33 out of 83 nuclear lncRNAs had at least three differentially expressed genes among their targets as defined by Hi-C **(S4A Table)**. The average effect of depletion of these 33 lncRNAs on the expression level of their target genes varied from -2.40 to 1.55 in log2 fold change (**S4B and S4C Table**). These genes were enriched for either upregulation or downregulation (combined P-value = 0.002; **Fig 3B; S5 Table**) demonstrating a concerted effect on target gene expression by the lncRNA knockdown. We found a 3.02 Mb average genomic distance between these 33 lncRNAs loci and their differentially expressed target genes, demonstrating they were not restricted to the immediate genomic neighborhood.

On average, 15.78% and 2.85% (P-value < 0.01) of the target genes of nuclear lncRNAs had a statistically significant positive and negative expression correlation, respectively **(S2 Fig)**. The higher number of positively correlated genes is in agreement with the general coexpression of genes near to each other on the genome [36]. Conversely, 81.34% of target genes were not significantly correlated with the lncRNA. This shows that expression correlation analysis and chromatin conformation analysis may yield different results due to technical limitations, to a complex relation between chromatin interaction and expression, or to lack of functionality of the lncRNA.

Using Gene Ontology (GO) enrichment analysis, we summarized the biological function of the identified target genes of the nuclear lncRNAs **(S6 and S7 Tables)**. As some lncRNAs facilitate the formation of condensates thought to assist in the recruitment of transcription factors [37], we performed transcription factor binding site (TFBS) motif enrichment analysis for the promoter regions of candidate target genes of each nuclear lncRNA. The number of lncRNAs with target genes enriched for at least one TFBS motif varied from 260 to 1,262 (22.12%-46.72%) among the selected cell types **(FDR** ≤ **0.1;** see **S8 and S9 Tables** for details). For most motifs (93.52% averaged across cell types), the TFBS occurrence in the promoter region of candidate target genes varied significantly between nuclear lncRNAs (chi-square test; P-values listed in **S10 Table**), indicating that different regulatory elements control the expression of candidate target genes of different lncRNAs.

### LncRNAs as potential recruiters of RBPs at candidate target genes

**Fig 4:**
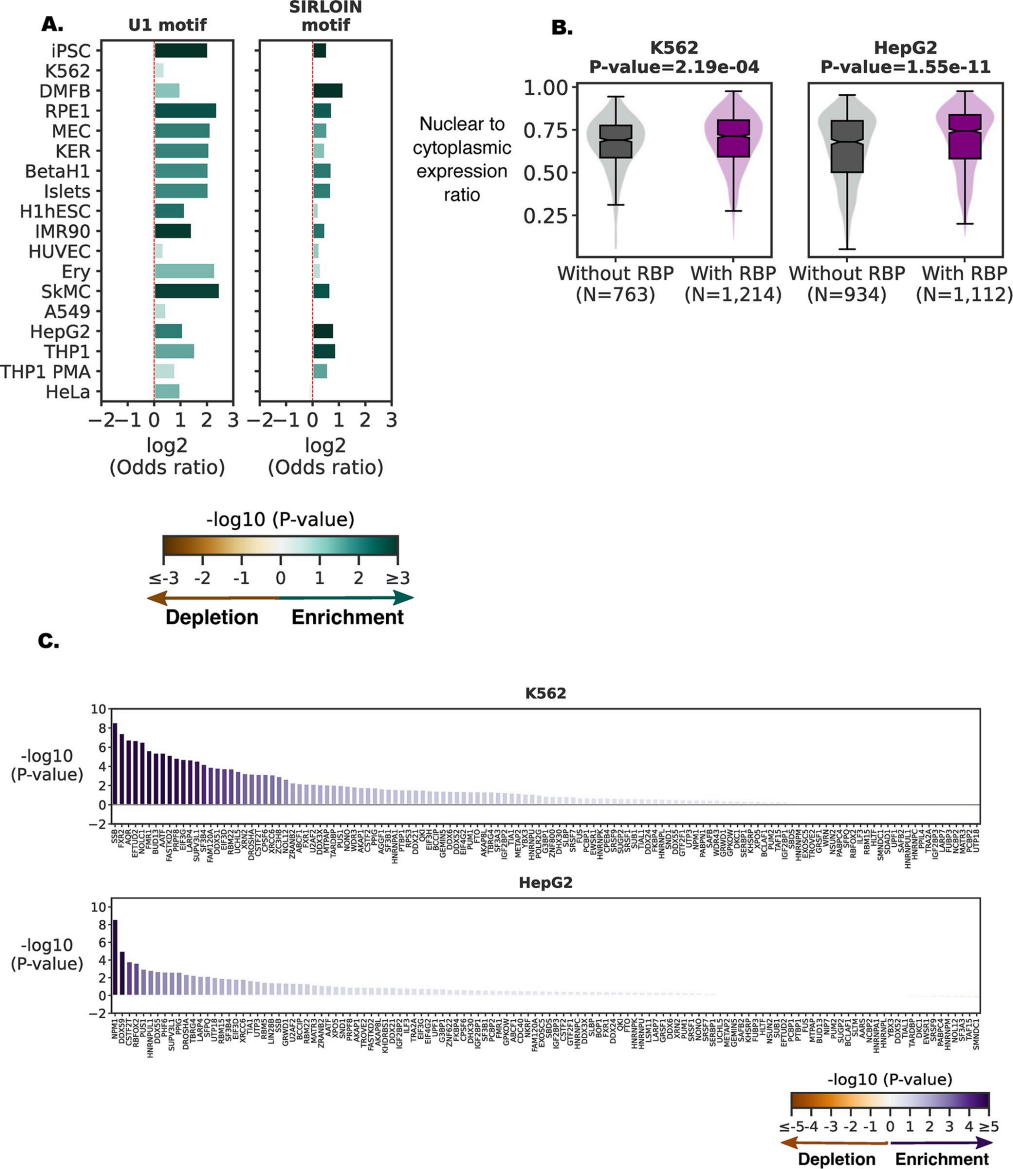
RBP binding nuclear lncRNAs. **(A)** Enrichment of lncRNAs with RNA features U1 repeat motif count and SIRLOIN motif count in the nuclear lncRNAs. Significance was calculated using a one-tailed Fisher’s exact test. (**B)** nuclear-to-cytoplasmic expression ratio distribution for lncRNAs with RBP binding in eCLIP data and lncRNAs without RBPs binding. Each panel shows one cell type for which RBP eCLIP data is available. The significance of the difference in nuclear-to-cytoplasmic expression ratio between two groups of lncRNAs was determined using a one-tailed Mann-Whitney U test. The cell type and P-value of significance are shown in the title. (**C)** Significance of enrichment of nuclear lncRNAs with RBP binding sites whose target genes are differentially expressed upon RBP knockdown compared to lncRNAs without binding sites for the RBP. Each panel shows one cell type. The y-axis shows -log10 (P-values) calculated using the one-tailed Fisher exact test. A positive value represents enrichment (Odds ratio >1), while the negative value (Odds ratio <1) represents depletion of lncRNAs with RBP binding sites.

**Fig 5:**
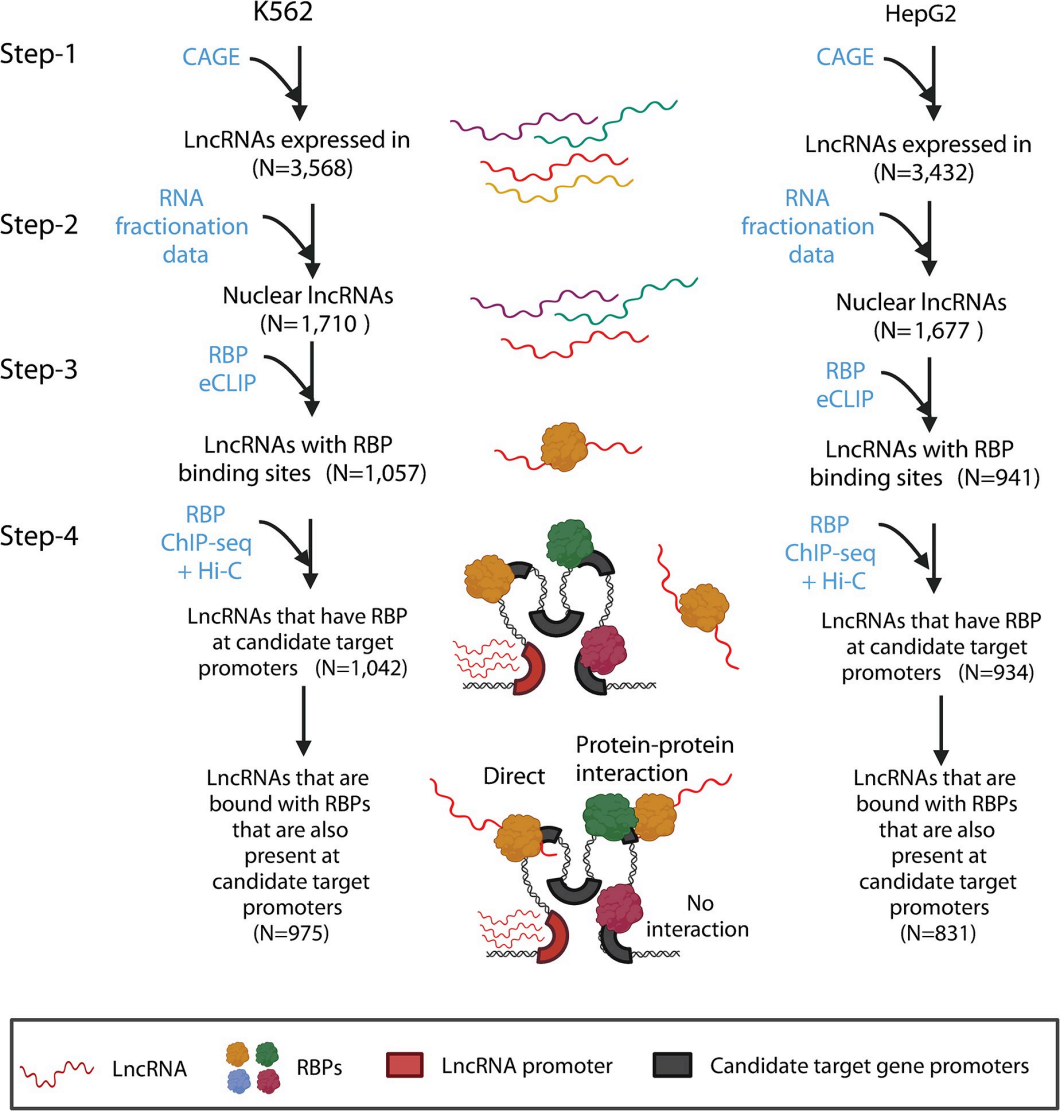
Steps to identify the nuclear lncRNAs that are bound to RBP that are also present at their candidate target gene promoters. The number of lncRNAs for each category is shown in parentheses. The data used for each step are shown in blue.

Some lncRNAs (e.g. DIGIT and XIST) have been shown to bind RBPs and facilitate their recruitment to chromatin [15–17]. The nuclear lncRNAs showed a significant enrichment for SIRLOIN and U1 sequences (**Fig 4A**), which are known to contain RBP binding sites [38,39]. Based on eCLIP (enhanced CLIP) data for K562 and HepG2 cells [40], RBP-bound lncRNAs had a significantly (P-value ≤ 2.2e-04) higher nuclear-to-cytoplasmic expression ratio than lncRNAs without any RBP interactions (**Fig 4B**), with 1,057 and 941 nuclear lncRNA transcripts in K562 and HepG2, respectively, bound by at least one RBP (**Fig 5**). For 56 out of 127 RBPs in K562 and 28 out of 122 RBPs in HepG2, genes differentially expressed after RBP silencing were significantly (P-value < 0.05) enriched for target genes of nuclear lncRNAs bound by these RBPs compared to target genes of lncRNAs not bound by the RBP (RNA-seq; **Figs 4C and 5; S11 Table**). We found that in general, an RBP tends to bind to promoters of genes targeted by lncRNAs bound by the RBP (**S12 Table;** K562 combined P-value 2.86e-29 and 12 out of 25 RBPs with P-value < 0.05, HepG2 combined P-value: 1.45e-09 and 6 out of 17 RBPs with P-value < 0.05). Further, protein-protein interaction data show that RBPs also tend to bind to promoters of genes targeted by lncRNAs bound by RBPs that can form protein-protein interaction with them (**S13 Table;** K562 combined P-value: 3.55e-20, HepG2 combined P-value: 5.65e-23). Together, this demonstrates that specific combinations of RBPs, lncRNAs, and promoters are interacting with each other.

**Fig 6:**
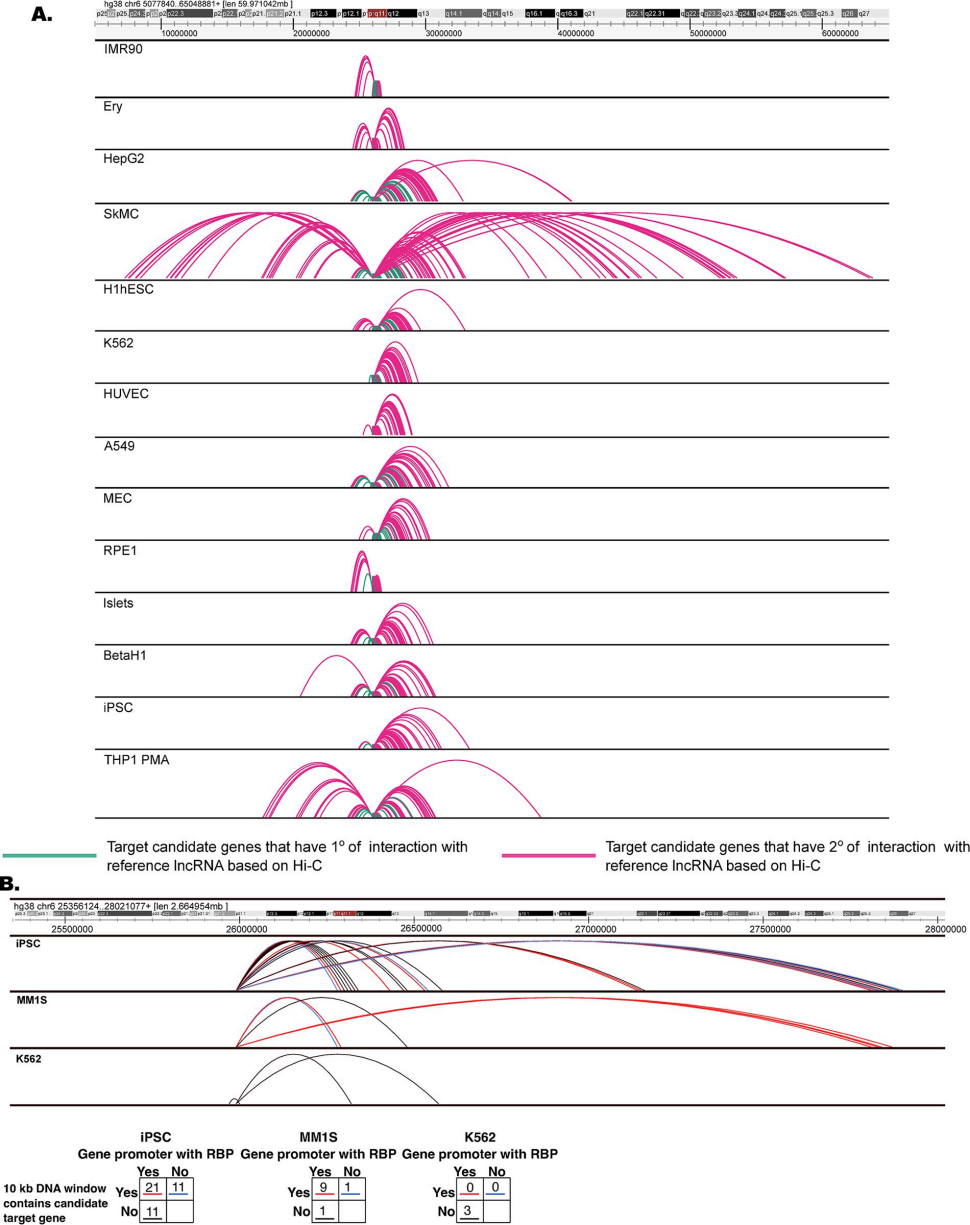
Hi-C and RNA-chromatin interactions for lncRNA ENSG00000272462. **(A)** The top track shows the genomic location of the interaction, followed by tracks showing the Hi-C annotated interactions between lncRNA ENSG00000272462 and its candidate target genes in different cell types. (**B)** RNA-chromatin interactions for lncRNAs in different cell types. The colors of interaction tracks for iPSC, MM1S and K562 cell types show RNA-chromatin interactions at regions with candidate target genes and presence or absence of RBPs at the promoter of these candidate targets in K562. The number of each type of interaction for all three cell types is shown in the tables below the figure.

As an example, we consider the currently unannotated broadly expressed lncRNA U91328.19 (ENSG00000272462) **(S3A Fig**) with nuclear-to-cytoplasmic expression ratio 0.54–0.85 (median 0.72) depending on cell type **(S3B Fig; Fig 6A; S1 Table; https://fantom.gsc.riken.jp/zenbu/reports/#Manuscript_examples)**. This lncRNA has been reported to be associated with GWAS SNPs related to hay fever and eczema [41]. GWAS heritability analysis showed that the A/B compartment containing this RNA gene is enriched for the trait ‘disease_ALLERGY_ECZEMA_DIAGNOSED’ **(S3C Fig)**. This is supported by the GO analysis, which showed enrichment for terms including ‘interleukin-7-mediated signaling pathway’, ‘innate immune response in mucosa’ and ‘antibacterial humoral response’ **(S3B Fig)**. In K562 and HepG2, ChIP-seq signals of HNRNPL, SRSF1, and ILF3 RBPs, involved in immune response pathways [42–45], were enriched at the candidate target gene promoters. eCLIP data showed that RBP HNRNPL, which interacts with ILF3, binds to the lncRNA in K562. Further, RNA-chromatin data for iPSC and MM1S showed that the lncRNA binds to the target regions bound by the RBPs HNRNPL, SRSF1, and ILF3 **(Fig 6B; S14 Table)**. These interactions were also observed in K562, but were not significant (significance of lncRNA forming a RNA-chromatin interaction FDR>0.01) possibly due to the lower sequencing depth of RNA-chromatin data in K562. Overall, this indicates that lncRNA U91328.19 may guide the recruitment of RBPs to the promoters of target genes involved in immune response.

### Interactive querying and visualization of lncRNA functional annotations across cell types

We created an interactive system, accessible at https://fantom.gsc.riken.jp/zenbu/reports/#F6_3D_lncRNA, to query and visualize the lncRNA properties, their candidate targets, and associations derived from our analysis, and to compare them across the 18 cell types. The visualization platform provides the analysis data for each lncRNA *viz*. 1) Genome browser view, 2) Genomic interactions, 3) Hi-C candidate gene targets and associated GO terms, TFBSs, RBPs, and genomic interactions.

As an example, the lncRNA HOTAIRM1 (ENSG00000233429) is expressed in multiple human cell types **(S4A Fig)** with nuclear-to-cytoplasmic expression ratio 0.55–0.86 depending on cell type. HOTAIRM1 regulates the local spatial arrangement of the HOXA gene clusters [46,47] and thereby the proliferation and differentiation of cells [47–49]. GO analysis of candidate targets shows enrichment for terms related to differentiation (**S4B and S5 Figs; https://fantom.gsc.riken.jp/zenbu/reports/#Manuscript_examples**). TFBSs for SREBF1,2, and SPZ1 transcription factors, associated with proliferation and differentiation [50–52], are enriched at the promoters of candidate targets of HOTAIRM1 **(S4C Fig)**. Consistent with previous work, HOTAIRM1 induction and short interfering RNA (siRNA) knockdown results in an increase and decrease in HOXA genes expression, respectively [46,47], and expression of the lncRNA and of HOXA cluster genes in the identified GO categories are positively correlated with each other (**S4B Fig)**. Knocking down HOTAIRM1 results in stronger chromatin interaction between HOX1/2 and HOX9/7 genes depending on the cell type [47]. Our analysis shows that expression of HOTAIRM1 is higher in Mammary epithelial cells (MEC) compared to H1hESC, while the genomic interactions between HOXA2 and HOXA9 are weaker (FDR ≤ 9.92e-9) in MEC compared to H1hESC. Overall, our analysis concurs with the previous findings [46,47], while revealing the cell type specific behavior of HOTAIRM1.

## Discussion

Nuclear lncRNAs are emerging as critical regulators in a wide variety of biological processes [7,18,53]. While several techniques have been developed that probe RNA-chromatin interactions [31,33,54–56], lncRNAs are underrepresented in these data due to their low expression levels compared to mRNAs. We show that lncRNA target regions determined using RNA-chromatin data are spatially proximal to the lncRNA gene, and therefore can be determined using Hi-C data. This is consistent with lncRNAs diffusing locally in the cell nucleus to find their genomic target sites. As a recent microscopy study showed that promoters are in close proximity but not necessarily in direct contact with their regulating enhancers [57], our analysis is based on spatial proximity without requiring direct interaction, thereby taking such indirect interactions and the mobility of lncRNAs in the nucleus into account. Knockdown of nuclear lncRNAs resulted in coherent differential expression patterns of their target genes, suggesting that cis-lncRNAs may have a regulatory role. However, since ASO knockdown effects are not fully consistent with those of other methods such as siRNAs [35], we cannot exclude the possibility of artifacts associated with the specific knockdown technology. Additionally, the transcriptome response may include indirect effects of the lncRNA knockdown.

Our analysis finds that predicted cis-lncRNAs may interact with RBPs enriched at the promoters of the lncRNA candidate target genes. LncRNAs may recruit RNA-binding proteins to form condensate-like structures [15,18,19] that assist in transcriptional regulation by controlling the concentration of transcription factors in a subnuclear region [37].

The number of nuclear lncRNAs ranged from 814 to 3,474 across the 18 cell types, and is affected by the availability of RNA subcellular fractionation expression data and specific protocol used. Cell type-specific lncRNAs may be missed for cell types for which RNA subcellular fractionation data are not available, while poly-A (minus) lncRNAs may be missed if only poly-A (plus) RNA data are available. The accuracy of the nuclear lncRNA identification depends on the quality and characteristics of the training data, such as the sequencing depth, in particular for very lowly expressed lncRNAs, and any occurrence of cross-contamination in the subcellular RNA sequencing data.

In contrast to RNA-based approaches, Hi-C is chromatin-based and does not directly depend on the expression level of the lncRNA. While exosome-sensitive lncRNAs are rapidly degraded and may therefore have a low prevalence in a cell, they may still be biologically relevant [27,58], underlining the importance of devising an annotation strategy independent of the expression level of the lncRNA.

Our analysis also does not depend on co-expression, an alternative method for predicting gene function, which is strongly affected by tissue composition, technical variation, and normalization issues that hinders the comparison of measured gene expression levels [59–64]. Importantly, it provides a single annotation of lncRNAs for all cell types, and therefore cannot identify cell type-specific lncRNA functions, whereas the Hi-C-based analysis provides target genes for each lncRNA in each cell type separately. Annotating lncRNAs based on the properties of genes in their genomic neighborhood is similarly unable to capture cell type-specific roles, and will fail altogether for intergenic lncRNAs with few or no annotated genes in their vicinity.

We systematically identified putative target regions of nuclear lncRNAs across 18 cell types and provide a web application to visualize these analysis results (https://fantom.gsc.riken.jp/zenbu/reports/#F6_3D_lncRNA). This resource can help to guide future experiments to discover lncRNA functions in mechanistic detail.

## Material and methods

### RNA-chromatin interactions

We collected and reprocessed published data from the following experiments: Red-C [34], and GRID-seq [33] (**S15 Table**) as described by Ryabykh *et al*. [65]. First, we applied FastUniq [66] for filtering out possible PCR duplicates in paired-end mode. Next, we used Trimmomatic [67] to detect and remove low-quality bases in paired-end mode with default parameters except for window size (5) and base quality threshold (26). Next, we excluded the read pairs lacking the presence of experiment-specific technical sequences to consider only proper RNA-DNA chimeric reads. Next, the RNA and DNA parts of contacts were collected in two separate files in fastq format. The RNA (longer than 13bp) and DNA (longer than 17bp) parts were independently mapped to the reference human genome (GRCh38.p13) with hisat2 [68].

The final list of RNA-DNA contacts included only the contacts with both DNA and RNA parts uniquely mapped to the reference genome with less than three mismatches; contacts with the RNA part mapping to splice junctions were discarded.

For all datasets, the resulting RNA part of the contacts corresponded to the reverse complementary strand of the respective RNA gene.

Red-C raw paired-end reads were processed as described in the original article [34]. The read pairs were pre-filtered based on the inclusion of the bridge segment in the 1st (forward) reads; for each contact, we obtained DNA, RNA-3`(neighboring the bridge sequence), and RNA-5`. We checked that the RNA-3’ and RNA-5’ parts are mapped to the opposite strands of the same chromosome within 10 kb from each other and considered only the RNA-3`part in the final list of contacts. As the GRID-seq data was already preprocessed by the authors (PCR duplicates were removed and technical bridge sequences were trimmed), the respective stages of our pipeline were skipped. Mapped RADICL-seq data from Yip *et al*. [32] was used for the analysis. Biorender.com was used to generate the S1A Fig.

### Identifying RNA-chromatin significant interactions

All genes (mRNA, intergenic, and non-intergenic lncRNA) that have at least one promoter with expression ≥ 0.5 TPM based on CAGE were used for the overlap analysis. The genome was divided into 10 kb bins and the annotated RNA reads were aggregated. To reduce the effect of genomic windows with very high interaction counts (typically found near the gene where the RNA is produced), skewing the distribution, we used an iterative approach in which significant interactions were removed from the data set in each iteration. The background probability for a bin was calculated by dividing the count of trans mRNA binding in that bin by the total number of trans mRNA reads. To estimate the significance of the RNA binding in each bin, we performed a one-sided binomial test using binom_test (x,n,p) from scipy where x = the number of reads for the gene in the bin; n = total number of remaining reads for the gene, p = background probability calculated using trans-binding mRNA in the bin. The binomial test was performed iteratively until no additional interactions (FDR ≤ 0.01) were found. For each iterative step, interactions with FDR ≤ 0.01 from the previous step were removed and the number of gene reads from those bins were subtracted from n (the total number of reads for the gene). The bins where gene interaction was supported by at least 3 reads and FDR ≤ 0.01 were defined as significant RNA-chromatin interactions. The genomic bins of significant RNA-chromatin interactions were annotated by mapping the strongest promoter for mRNAs, intergenic lncRNAs, and non-intergenic lncRNAs to identify RNA-chromatin gene pairs.

### Subcellular fractionation expression

The CAGE fractionation data for cell types iPSC, DMFB, A549, H1hESC, HUVEC, HeLa, HepG2, IMR90, and K562 (**S16 Table**) and was used to calculate the expression of each gene in the nuclear and cytoplasmic fractions (TPM). The formula used to calculate the nuclear-to-cytoplasmic expression ratio is (*nuclear expression*)/(*nuclear expression + cytoplasmic expression*). In case of iPSC and DMFB nuclear-to-cytoplasmic expression ratio was calculated using formula *mean (chromatin expression*, *nucleoplasm expression) / [mean (chromatin expression*, *nucleoplasm expression) + (cytoplasmic expression)]*. For the cell types where fractionation expression data was not available, the fractionation values averaged over cell types were assigned.

### Sequence features

All the exons from the transcripts associated with the expressed promoters were selected to search for the sequence features. The SIRLOIN motif representative motif [CT][GA]CCTCCC[GA][GA]GTTCAAG[CT]GAT[TC]CTCCT[GA]CCTCAGCCTCCCGA, obtained from [39] and the U1 motif representative sequence CAGGTGAGT were searched in the selected exons using function fuzznuc of EMBOSS package.

### Hi-C data generation and processing

iPSC Hi-C data was generated as described in Ramilowski *et al*. [35] while data for the remaining cell types were obtained from previously published studies (**S3 and S17 Tables**). Data for each replicate was processed using HICUP ver. 0.5.10 [69] which involved read truncation, mapping, filtering experimental artifacts, and de-duplication. The alignment files for all replicates for each cell type were merged to perform the downstream analysis. GRCh38 primary human genome assembly (hg38) was used for the analysis.

### Gene and enhancer models and primary annotations

FANTOM CAT gene models [1] and hg38 FANTOM 5 bidirectional enhancers [70] (enhancers) (https://zenodo.org/record/556775) were used as the primary genome annotation. The FANTOM CAT gene classes used in this study are mRNAs (protein-coding), intergenic lncRNAs, antisense lncRNAs, divergent lncRNAs, and sense intronic lncRNAs, with the latter three classes collectively referred to as non-intergenic lncRNAs.

### The expression level of promoters and bi-directional enhancers

Expression levels of promoters and enhancers were determined using CAGE data. iPSC CAGE data was generated for this study using the nAnT-iCAGE protocol [71], while publicly available matched CAGE libraries were used for the remaining cell types **(S18 Table)**. Promoter CAGE tag counts were estimated by intersecting CTSS files for individual libraries with the promoter bed file using bedtools (ver. 2.26.0) [72] and were normalized to calculate promoter expression in tags per million (tpm). The expression for a promoter in a cell type was determined by calculating the mean expression across all CAGE libraries for that cell type. For each gene, the promoter with the highest expression level, requiring a minimum expression of 0.5 tpm, and minimum 3 tag counts in at least one CAGE library was used for the downstream analysis. Next, the CAGE expression of enhancers was calculated by summing the CAGE tag counts across the libraries for each cell type, ignoring the tag directionality. All enhancers with an aggregate tag count of at least 5 were used for the downstream analysis.

In the absence of matched CAGE libraries, FANTOM5 data for pancreatic tissues was repurposed for Islets and BetaH1 cells. First, expressed transcripts in Islets and BetaH1 cells were determined using RNA-seq data from published studies [73,74] (**S18 Table**) using Kallisto ver. 0.45.0 [75]. Next, the strongest promoter for each gene was determined based on expressed transcripts in each cell type and pancreatic tissue CAGE data. Expressed enhancers in pancreatic tissues were assigned to both Islets and BetaH1 cell types.

### Promoter types

The precalculated chromatin state ChromHMM models for the selected cell types were downloaded from the previous studies listed in **S18 Table**. The strongest promoters were intersected with corresponding cell type chromatin state models and were assigned a promoter type: H3K4me3 enriched (canonical promoters), H3K4me1 enriched (enhancer-like promoters), or Neither (undetermined) depending on the overlapping state **(S18 Table)**. As chromatin state data were not available for THP1, THP1-PMA, and RPE-1, promoter types provided by FANTOM-CAT [1] were used to annotate promoters in these cell types **(S18 Table)**.

### A/B compartments, TADs, and loops

Hi-C alignment.bam files for individual replicates and merged data were converted into.hic format using an in-house awk script and the Pre command from the Juicer package [76]. A/B compartments were identified at 1 Mb resolution using the function eigenvector from Juicer package with options: -p VC <hic file> <chromosome name> BP 1000000. A positive eigenvalue represents compartment A (transcriptionally active compartment) and a negative eigenvalue represents compartment B (a compartment with lower transcriptional activity compared to compartment-A). The compartments were redefined by reassigning the signs (+/-) to eigenvalues in cases where average expression values (determined using CAGE peaks) of compartment B (negative value) were higher than those of compartment A (positive value). Further, TADs and chromatin loops were calculated as a resource for the research community using functions from the Juicer package.

### Determining significant genomic interactions

The intra-chromosomal genomic interactions were identified using the Bioconductor package GOTHiC [77]. The Hi-C data across biological replicates for each cell type was merged and statistically significant cis-genomic interactions were identified at 10 kb resolution. The alignment.bam files were converted into.gothic files using the format conversion script hicup2gothic from the HiCUP package. The interactions were calculated using.gothic together with corresponding restriction enzyme files **(S3 Table)** for each cell type. All the interactions supported by at least 5 read pairs and q-value ≤ 0.05 were defined as significant genomic interactions. The number of genomic interactions per cell type varied from 2,540,361 to 46,975,256 with Hi-C sequencing depth (**S19 Table**) and included interactions in both A and B compartments (compartment-A are genomic regions with higher transcription activity compared to compartment-B genomic regions) (**S19 Table**). The interactions were annotated by overlaying the expressed promoters and enhancers in the selected cell types to identify interacting promoters. In cases where promoters for more than one gene overlapped the same 10 kb region, the interactions were counted multiple times, with one interaction for each gene. Interactions with annotations on both sides were used for the downstream pairwise analysis. The number of annotated cis-interactions varied from 50,170 to 604,677 **(S19 Table)** among the cell types.

### Differential Hi-C interaction analysis

An interaction read count table (10 kb resolution) for individual Hi-C replicates was generated using straw (ver. 0.0.8). Islets and BetaH1 data were excluded from the analysis as they had only one Hi-C library. The pairwise differential Hi-C analysis was performed using the Bioconductor package multiHiCcompare (ver. 1.8.0) [78]. All genomic interactions with 5 read counts in at least two Hi-C libraries were tested for the differential interactions. Interactions with **|**log2(Fold change)**|** ≥ 1 and FDR-corrected P-value ≤ 0.1 were defined as differential Hi-C interactions.

### Hi-C target genomic regions and genes

Target genomic regions for each lncRNA were defined using significant genomic interactions. The genomic window overlapping with the reference lncRNA promoter was selected as the reference genomic region. All genomic regions connected to the reference genomic region by Hi-C interaction up to 2 degrees of Hi-C interactions within the A or B compartment (extended by +/- 100 kb) in which the reference lncRNA regions is situated were used to define the target genomic regions **(Fig 3A)**. The expressed promoters and enhancers were mapped to the target genomic regions to identify the potential target genes **(Fig 3A).**

### Enrichment of differentially expressed genes in the target genes of lncRNAs due to ASO knockdown of predicted cis-lncRNAs

The differentially expressed (DE) targets genes due to the knockdown of 64 predicted cis-lncRNA were identified using precalculated DE genes provided by Ramilowski *et al*. (https://fantom.gsc.riken.jp/6/suppl/Ramilowski_et_al_2020/data/DEGs/) [35]. DE genes with FDR-corrected P-value ≤ 0.1 in at least one ASO were included for the analysis. The significance of differentially expressed genes among the nuclear lncRNA target genes was calculated using a one-sided Fisher’s exact test. The contingency table used for analysis is described in **S20 Table**.

### Gene ontology (GO) enrichment analysis

The GO term database from NCBI was downloaded on Nov 28^th^, 2019 (file: gene2go from http://ftp.ncbi.nlm.nih.gov/gene/DATA/). All mRNA genes with an entrezID that are expressed in a given cell type and are target genes for at least one lncRNA were used for the analysis. The GO term enrichment analysis for each lncRNA target gene was performed using a one-sided Fisher’s exact test (details are given in **S21 Table**). The background consisted of all target genes for all expressed lncRNAs other than the target genes of the reference lncRNA. The analysis was performed for the GO terms that have at least one mRNA gene in their geneset in common with the lncRNA target genes list. The P-value was corrected for multiple testing using the Benjamini–Hochberg false discovery rate (FDR) multiple testing correction method. All GO terms with FDR-adjusted P-value ≤ 0.1 and at least 3 mRNA genes in their geneset in common with the lncRNA’s target genes were defined as significant GO terms. Further, GO enrichment analysis was also performed for gene sets provided by the Broad Institute [79–81] and EnrichR gene sets (downloaded on March 4^th^, 2021 from https://maayanlab.cloud/Enrichr/#stats) [82] as a resource for annotating the lncRNA target genes.

### Hi-C target gene expression correlation analysis

For each lncRNA, the Spearman correlation was calculated for gene expression between the lncRNA and their target mRNA genes across the 18 cell types. To determine if the lncRNA’s target genes have a preference for genes that have positive or negative expression correlation with the reference lncRNA, a one-sample Student’s t-test was performed. The lncRNAs with target genes with P-value ≤ 0.05, and t-statistic value positive or negative were categorized as lncRNAs with positive expression correlation or negative expression correlation respectively, or otherwise with no preference. The analysis was performed in the same manner including only GO-annotated mRNA genes.

### TFBSs enrichment analysis and motif correlation

Genome-wide TFBS predictions for SwissRegulon motifs [83–85] were downloaded for the hg38 human genome assembly. For each cell type, the predicted TFBSs were intersected with promoter and enhancer regions extended by +/- 250 bp. In cases where multiple TFBSs for the same motif overlapped with a promoter or enhancer, the posterior probability scores of the predicted TFBSs were summed. The TFBSs with aggregate scores of at least 0.1 were assigned to the promoters and enhancers. Motifs with predicted TFBSs overlapping with less than 200 promoters and enhancers were excluded from the analysis. The significance of motif enrichment in the lncRNA target genes was calculated using a one-sided Fisher’s exact test. The contingency table used for analysis is described in **S22 Table**. The P-values were corrected for multiple testing using the Benjamini–Hochberg approach. All lncRNAs with at least 5 target genes (promoters and enhancers) were tested for enrichment. Fisher combined P-value for every lncRNA (combining over the motifs) in each cell type was calculated to identify the lncRNAs with target gene promoters and enhancers significantly enriched for TFBSs.

### RBP enrichment analysis

Overview and analysis of ENCODE eCLIP data (**S23 Table**): ENCODE eCLIP data for cell lines HepG2 and K562 was used for the analysis. In total, data for 234 <RBP, cell type> pairs were analyzed as follows: (1) raw reads were preprocessed as in the original eCLIP pipeline[86], (2) trimmed reads were mapped to the hg38 genome assembly with F6 CAT genome annotation using hisat2[68], (3) the aligned reads were deduplicated [84] and the uniquely mapped and correctly paired reads were filtered with samtools [87], (4) gene-level total read counts in exons were obtained with plastid [88] for CLIP and control samples, (6) comparison of gene-level CLIP read counts against matched controls was performed using edgeR [89] following the standard differential expression analysis pipeline. Based on (6), reliable RNA targets of each RBP were defined as those passing 5% FDR and log_2_FC > 0.5. RBPs with fewer than 15 lnRNA target genes were excluded from the downstream analysis resulting in the final set of 207 <RBP, cell type> pairs.

Overview and analysis of ENCODE ChIP-Seq data: Optimally thresholded ChIP-Seq peaks of 18 and 26 RBPs for HepG2 and K562, respectively were downloaded from ENCODE and merged across replicates. U2AF1 (in K562) was excluded from the analysis as having less than 15 RNA targets in eCLIP data. To annotate promoters and enhancers with ChIP-Seq peaks, we required non-zero overlap between the respective genomic regions and peaks.

*Analysis of lncRNA Hi-C gene targets enrichment with RBP targets*: Right-tailed Fisher’s exact test (on promoters and enhancers) was used to identify lncRNA gene targets enriched with RBP targets. Benjamini-Hochberg (FDR) correction for multiple tested RBPs was applied and cases passing FDR adjusted P-value ≤ 0.1 and with at least 3 RBP targets were considered statistically significant.

### RBP protein-protein interaction

Protein-protein interaction map for K562 and HepG2 RBPs was generated using STRING (https://string-db.org/; ver. 11.5). Parameters used to identify the interactions are: Network type: “full STRING network”, Meaning of network edges: “evidence”, and active interaction sources: “Textmining, Experiments, Databases, Neighborhood, Gene Fusion and Co‑occurrence”. Protein-protein interactions with score >0.4 were used for the downstream analysis.

### Heritability enrichment analysis

We used stratified linkage disequilibrium (LD) score regression (ldsc software ver. 1.0.0, https://github.com/bulik/ldsc) [90] to partition the common (minor allele frequency (MAF) > 5% in European 1000 Genomes Project Phase 3 data) SNP heritability for 47 UK Biobank traits and diseases (https://data.broadinstitute.org/alkesgroup/UKBB), and four diseases analyzed by O’Connor *et al*. [91–95]. We tested partitioned heritability of each Hi-C annotation (converted to hg19 genome build using UCSC liftOver tool) conditioning on the baselineLD model ver. 2.2 (https://data.broadinstitute.org/alkesgroup/LDSCORE/1000G_Phase3_baselineLD_v2.2_ldscores.tgz). We calculated the significance of the regression coefficient for the Hi-C annotation using the Z-score.

### Enrichment analysis for differentially expressed predicted cis-lncRNA target genes due to RBP knockdown

ENCODE RNA-seq data for K562 and HepG2 after RBP silencing using shRNA were used to compare predicted cis-lncRNA with and without RBP binding sites [96]. The list of differentially expressed genes due to RBP knockdown was downloaded from the ENCODE data portal. All genes with FDR-corrected P-value ≤ 0.1 were considered as DE genes. Based on eCLIP data, RBPs that bind up to 500 lncRNAs were considered for the analysis. One-tailed Fisher’s exact test was used to identify the RBPs for which knockdown resulted in differential expression of the lncRNA target genes. Biorender.com was used to generate the RBP interaction figure.

## Supporting information

S1 Fig Number of RNA-chromatin interactions in different cell types. **(A)** Schematic diagram showing the steps used to calculate the significant RNA-chromatin interactions. (**B)** Number of intra-chromosomal and inter-chromosomal significant RNA-chromatin interactions for nuclear lncRNAs in different cell types. The technology used to generate the RNA-chromatin data is shown in parentheses next to the cell type name. (**C)** Number of nuclear lncRNAs with intra-chromosomal and inter-chromosomal significant interactions in different cell types.
(TIF)

S2 Fig Percentage of target genes with significant expression correlation with its reference lncRNA. Each panel corresponds to one cell type and shows the percentage of all the targets with significant (P-value ≤ 0.01) positive (red) and negative (blue) expression correlation with reference lncRNA. The cell type’s name is shown in each panel’s title.
(TIF)

S3 Fig Annotation results for lncRNA ENSG00000272462. **(A)** Expression of the lncRNA in all 17 cell types (CAGE data from pancreas were used for both BetaH1 and islet cells). **(B)** GO annotation results. The topmost heatmap shows the expression correlation between the lncRNA and GO-annotated genes in the candidate target genes. For readability, the gene names are shown on the top of the heatmap in the same order as in the heatmap. The middle heatmap shows whether the candidate target genes in each cell contain each of the GO-annotated genes. The bottom heatmap shows the membership of each GO gene in each GO category, and the heatmap on the right shows whether each GO term is enriched in each cell type. **(C)** GWAS trait enriched (FDR adjusted P-value ≤ 0.1) in the A/B compartment overlap with the lncRNA’s candidate target genes. **(D)** Motifs enriched (FDR adjusted P-value ≤ 0.1) in the lncRNA’s candidate target genes.
(TIF)

S4 Fig Annotation results for lncRNA ENSG00000233429. **(A)** Expression of the lncRNA in all 17 cell types (CAGE data from pancreas were used for both BetaH1 and islet cells). **(B)** GO annotation results. The topmost heatmap shows the expression correlation between the lncRNA and GO-annotated genes in the candidate target genes. For readability, the gene names are shown on the top of the heatmap in the same order as in the heatmap. The middle heatmap shows whether the candidate target genes in each cell contain each of the GO-annotated genes. The bottom heatmap shows the membership of each GO gene in each GO category, and the heatmap on the right shows whether each GO term is enriched in each cell type. **(C)** Motifs enriched (FDR adjusted P-value ≤ 0.1) in the lncRNA’s candidate target genes. **(D)** GWAS trait enriched (FDR adjusted P-value ≤ 0.1) in the A/B compartment overlap with the lncRNA’s candidate target genes.
(TIF)

S5 Fig Hi-C and RNA-chromatin interactions for lncRNA ENSG00000233429. The top track shows the genomic location of the interaction, followed by tracks showing the Hi-C annotated interactions between lncRNA ENSG00000233429 and its candidate target genes in different cell types.
(TIF)

S1 Table Nuclear-to-cytoplasmic expression ratio for each lncRNA in individual cell types. (XLSX)

S2 Table GWAS traits significantly enriched the A/B compartment of each cell type. (XLSX)

S3 Table Details of Hi-C data per cell type used for the study. (XLSX)

S4 Table Differentially expressed genes after knockdown of nuclear lncRNAs in dermal fibroblast. (XLSX)

S5 Table Enrichment of upregulated or downregulated genes in candidate target genes as result of lncRNA knockdown in dermal fibroblast. (XLSX)

S6 Table The number of nuclear intergenic and non-intergenic nuclear lncRNAs with at least one GO term enriched in their target genes. (XLSX)

S7 Table GO terms significantly enriched in target genes of the lncRNAs in each cell type. (XLSX)

S8 Table The number of nuclear intergenic and non-intergenic lncRNAs with at least one TFBSs enriched in their target genes. (XLSX)

S9 Table TFBS motifs significantly enriched in the Hi-C gene targets of lncRNAs in each cell type. (XLSX)

S10 Table Chi-squared test P-value for each TFBS motif in every cell type. (XLSX)

S11 Table Enrichment of differentially expressed genes in candidate target genes as result of knockdown of RBPs that are bound to nuclear lncRNAs. (XLSX)

S12 Table Significance (Fisher’s exact test) of RBP binds to promoters of genes targeted by nuclear lncRNAs bound by the same RBP. (XLSX)

S13 Table Significance (Fisher’s exact test) of RBP binds to promoters of genes targeted by nuclear lncRNAs bound by the RBP that interacts with promoter RBP. (XLSX)

S14 Table Details of RNA-chromatin interactions at genomic window with ENSG00000272462 candidate target genes with RBP binding at their promoter. (XLSX)

S15 Table Overview of the reprocessed RNA-chromatin data. (XLSX)

S16 Table Details of subcellular RNA fractionation data. (XLSX)

S17 Table Mapping statistics for Hi-C data. (XLSX)

S18 Table Details of CAGE data and ChromHMM states used to annotate the promoters. (XLSX)

S19 Table Genomic interactions at 10 kb resolution. (XLSX)

S20 Table Contingency table for differentially expressed genes enrichment analysis. (XLSX)

S21 Table Contingency table for GO enrichment analysis. (XLSX)

S22 Table Contingency table for motif enrichment analysis. (XLSX)

S23 Table List of RBPs used for the analysis. (XLSX)
