# Correction: Annotation of nuclear lncRNAs based on chromatin interactions

**DOI:** 10.1371/journal.pone.0339624

**Published:** 2026-04-28

**Authors:** Saumya Agrawal, Andrey Buyan, Jessica Severin, Masaru Koido, Tanvir Alam, Imad Abugessaisa, Howard Y. Chang, Josée Dostie, Masayoshi Itoh, Juha Kere, Naoto Kondo, Yunjing Li, Vsevolod J. Makeev, Mickaël Mendez, Yasushi Okazaki, Jordan A. Ramilowski, Andrey I. Sigorskikh, Lisa J. Strug, Ken Yagi, Kayoko Yasuzawa, Chi Wai Yip, Chung Chau Hon, Michael M. Hoffman, Chikashi Terao, Ivan V. Kulakovskiy, Takeya Kasukawa, Jay W. Shin, Piero Carninci, Michiel J. L. de Hoon

There are errors in the author affiliations. The correct affiliations are as follows:

Saumya Agrawal^1^, Andrey Buyan^2,3^, Jessica Severin^1^, Masaru Koido^1,4^, Tanvir Alam^5^, Imad Abugessaisa^1^, Howard Y. Chang^6^, Jose´e Dostie^7^, Masayoshi Itoh^1,8^, Juha Kere^9,10^, Naoto Kondo^11^, Yunjing Li^12^, Vsevolod J. Makeev^3^, Mickael Mendez^13^, Yasushi Okazaki^1^, Jordan A. Ramilowski^1,14^, Andrey I. Sigorskikh^3^, Lisa J. Strug^12,13,15,16^, Ken Yagi^1^, Kayoko Yasuzawa^1^, Chi Wai Yip^1^, Chung Chau Hon^1^, Michael M. Hoffman^13,17,18,19^, Chikashi Terao^1^, Ivan V. Kulakovskiy^2,3^, Takeya Kasukawa^1^, Jay W. Shin^1,20^, Piero Carninci^1,21^, Michiel J. L. de Hoon^1^

**1** RIKEN Center for Integrative Medical Sciences, Yokohama, Japan, **2** Autosome.org, **3** FANTOM Consortium, **4** Institute of Medical Science, The University of Tokyo, Tokyo, Japan, **5** College of Science and Engineering, Hamad Bin Khalifa University, Doha, Qatar, **6** Center for Personal Dynamic Regulome, Stanford University, Stanford, California, United States of America, **7** Department of Biochemistry, Rosalind and Morris Goodman Cancer Research Center, McGill University, Montre´al, Que´bec, Canada, **8** RIKEN Preventive Medicine and Diagnosis Innovation Program, Wako, Japan, **9** Department of Biosciences and Nutrition, Karolinska Institutet, Huddinge, Sweden, **10** Stem Cells and Metabolism Research Program, University of Helsinki and Folkhälsan Research Center, Helsinki, Finland, **11** RIKEN Center for Life Science Technologies, Yokohama, Japan, **12** Division of Biostatistics, Dalla Lana School of Public Health, University of Toronto, Toronto, Ontario, Canada, **13** Department of Computer Science, University of Toronto, Toronto, Ontario, Canada, **14** Advanced Medical Research Center, Yokohama City University, Yokohama, Japan, **15** Department of Statistical Sciences, University of Toronto, Ontario, Canada, **16** The Centre for Applied Genomics and Program in Genetics and Genome Biology, The Hospital for Sick Children, Toronto, Ontario, Canada, **17** Princess Margaret Cancer Centre, Toronto, Ontario, Canada, **18** Department of Medical Biophysics, University of Toronto, Toronto, Ontario, Canada, **19** Vector Institute, Toronto, Ontario, Canada, **20** Genome Institute of Singapore (GIS), Agency for Science, Technology and Research (A*STAR), Singapore, Republic of Singapore, **21** Human Technopole, Milan, Italy
