# Supplementary figures and images for: Annotation of nuclear lncRNAs based on chromatin interactions
Source: PLoS One. 2024 May 6;19(5):e0295971. doi: 10.1371/journal.pone.0295971 (PMC11073715; doi:10.1371/journal.pone.0295971)

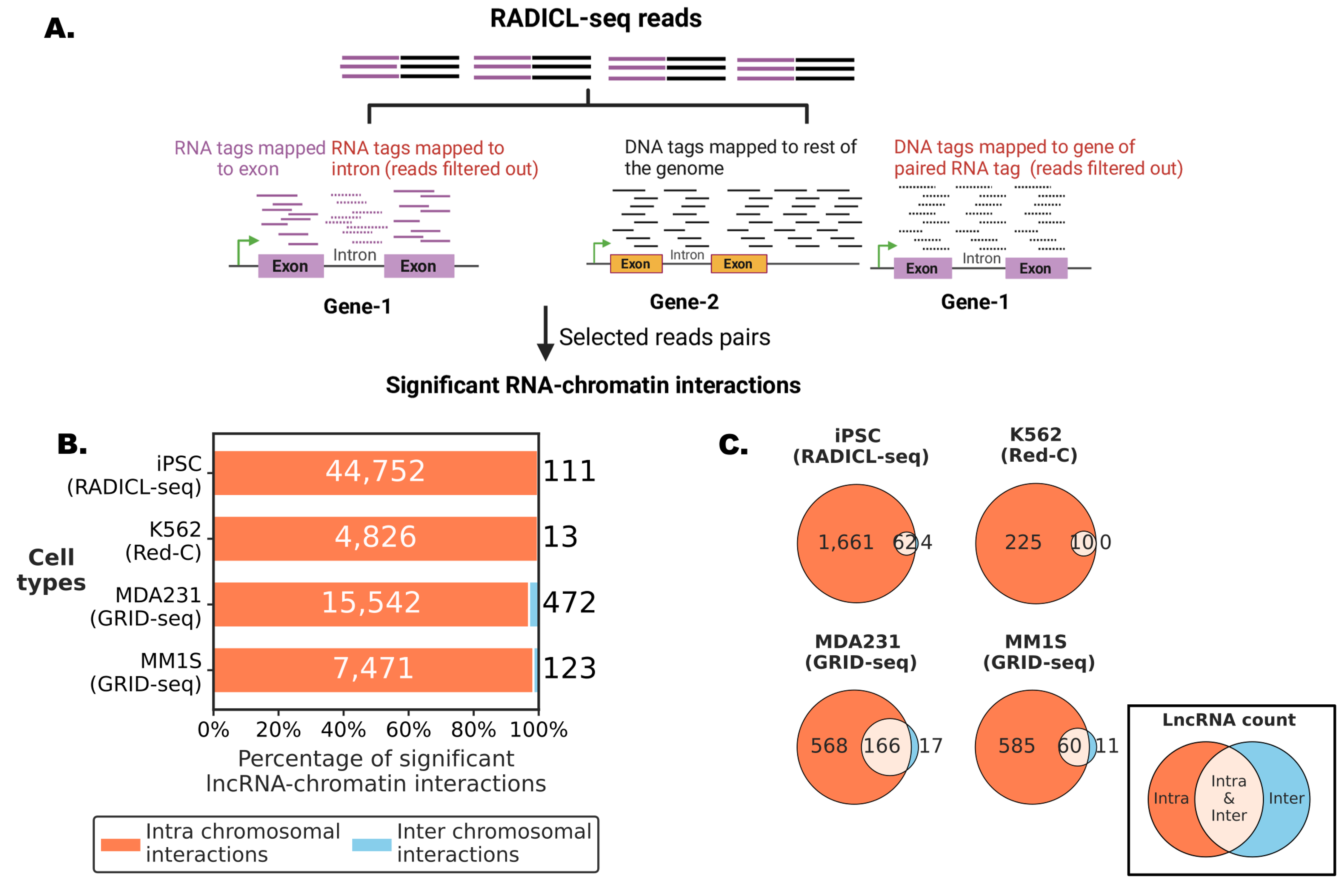

Supplement: S1 Fig — (A) Schematic diagram showing the steps used to calculate the significant RNA-chromatin interactions. (B) Number of intra-chromosomal and inter-chromosomal significant RNA-chromatin interactions for nuclear lncRNAs in different cell types. The technology used to generate the RNA-chromatin data is shown in parentheses next to the cell type name. (C) Number of nuclear lncRNAs with intra-chromosomal and inter-chromosomal significant interactions in different cell types. (TIF) [file pone.0295971.s001.tif]

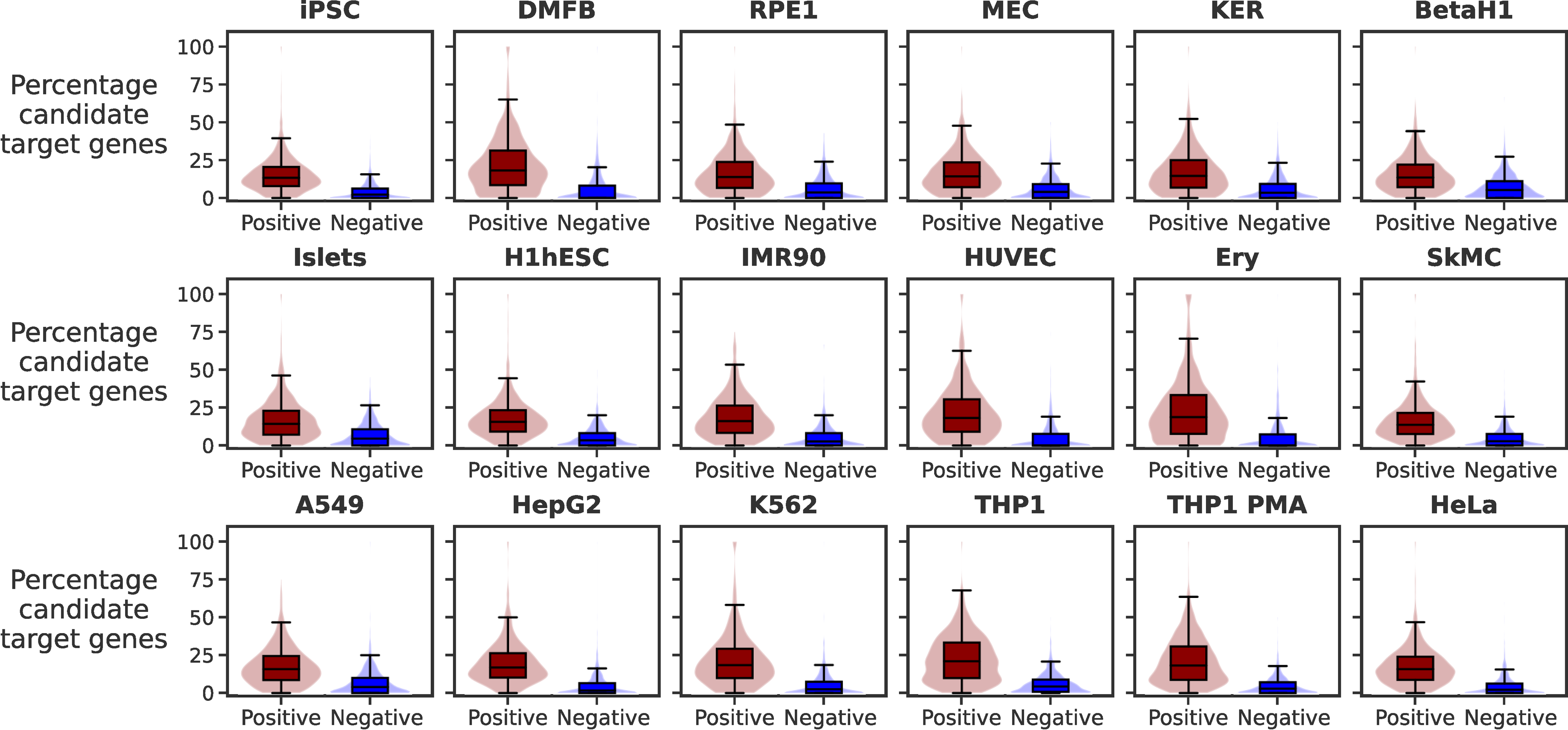

Supplement: S2 Fig — Each panel corresponds to one cell type and shows the percentage of all the targets with significant (P-value ≤ 0.01) positive (red) and negative (blue) expression correlation with reference lncRNA. The cell type’s name is shown in each panel’s title. (TIF) [file pone.0295971.s002.tif]

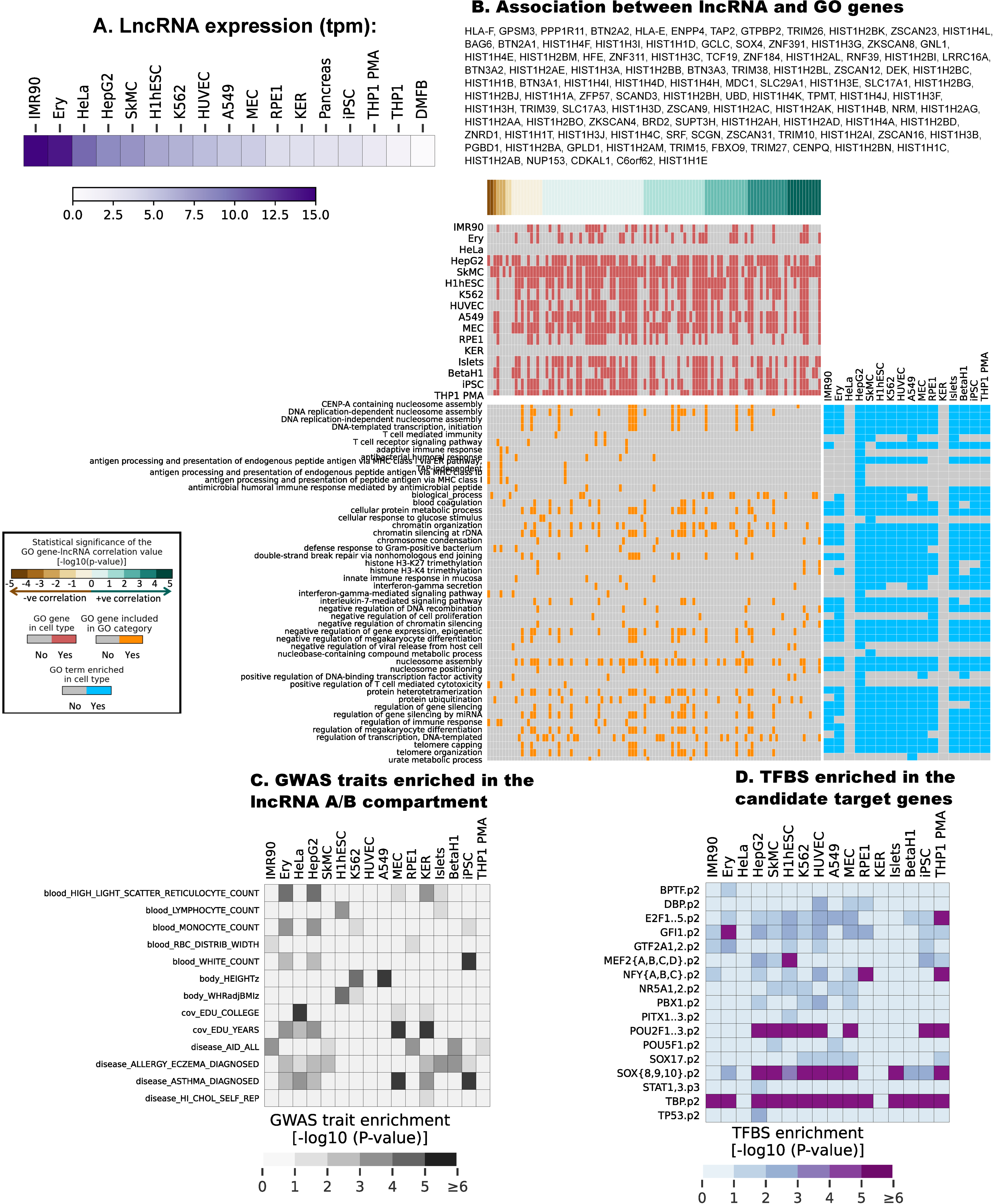

Supplement: S3 Fig — (A) Expression of the lncRNA in all 17 cell types (CAGE data from pancreas were used for both BetaH1 and islet cells). (B) GO annotation results. The topmost heatmap shows the expression correlation between the lncRNA and GO-annotated genes in the candidate target genes. For readability, the gene names are shown on the top of the heatmap in the same order as in the heatmap. The middle heatmap shows whether the candidate target genes in each cell contain each of the GO-annotated genes. The bottom heatmap shows the membership of each GO gene in each GO category, and the heatmap on the right shows whether each GO term is enriched in each cell type. (C) GWAS trait enriched (FDR adjusted P-value ≤ 0.1) in the A/B compartment overlap with the lncRNA’s candidate target genes. (D) Motifs enriched (FDR adjusted P-value ≤ 0.1) in the lncRNA’s candidate target genes. (TIF) [file pone.0295971.s003.tif]

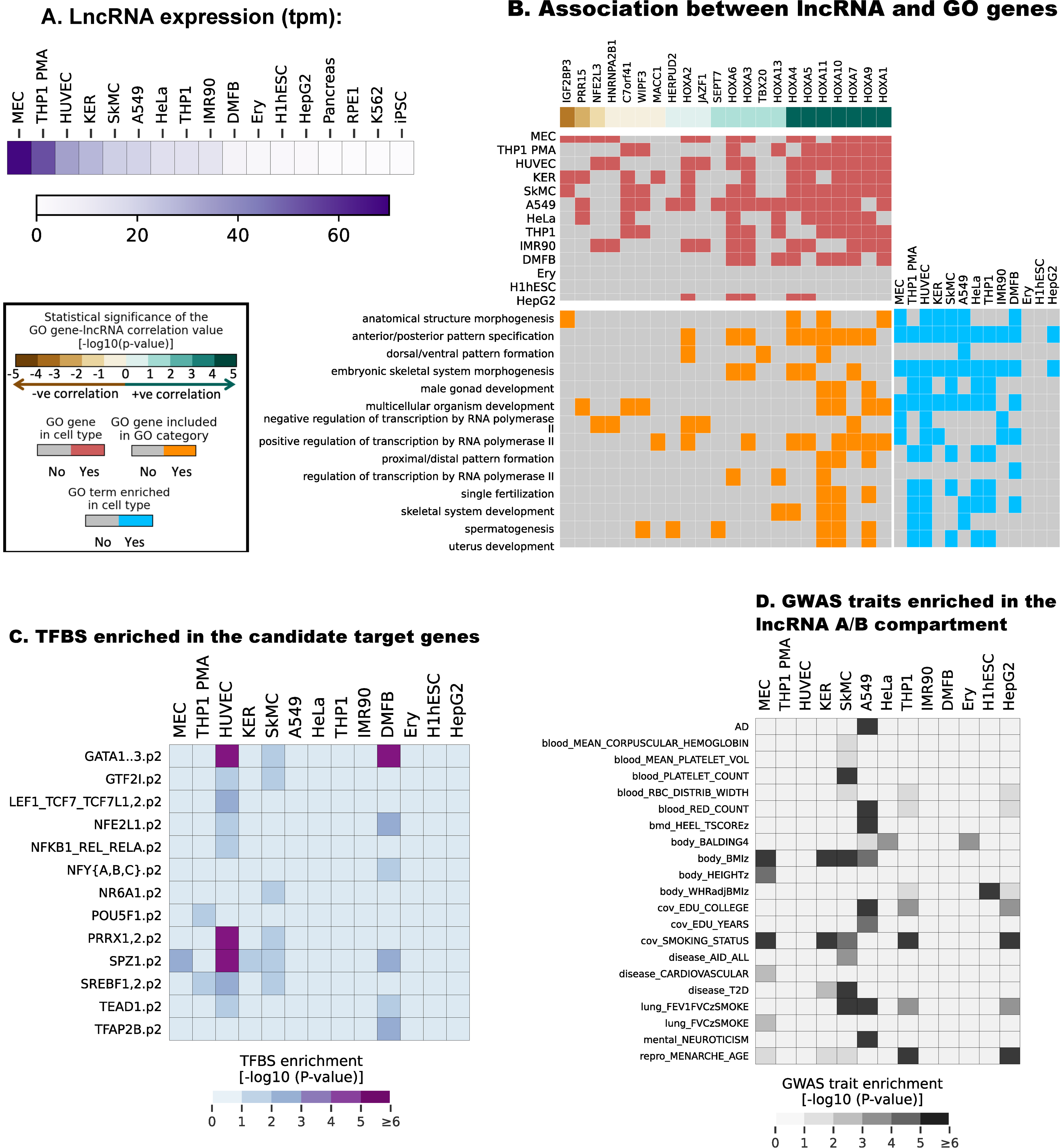

Supplement: S4 Fig — (A) Expression of the lncRNA in all 17 cell types (CAGE data from pancreas were used for both BetaH1 and islet cells). (B) GO annotation results. The topmost heatmap shows the expression correlation between the lncRNA and GO-annotated genes in the candidate target genes. For readability, the gene names are shown on the top of the heatmap in the same order as in the heatmap. The middle heatmap shows whether the candidate target genes in each cell contain each of the GO-annotated genes. The bottom heatmap shows the membership of each GO gene in each GO category, and the heatmap on the right shows whether each GO term is enriched in each cell type. (C) Motifs enriched (FDR adjusted P-value ≤ 0.1) in the lncRNA’s candidate target genes. (D) GWAS trait enriched (FDR adjusted P-value ≤ 0.1) in the A/B compartment overlap with the lncRNA’s candidate target genes. (TIF) [file pone.0295971.s004.tif]

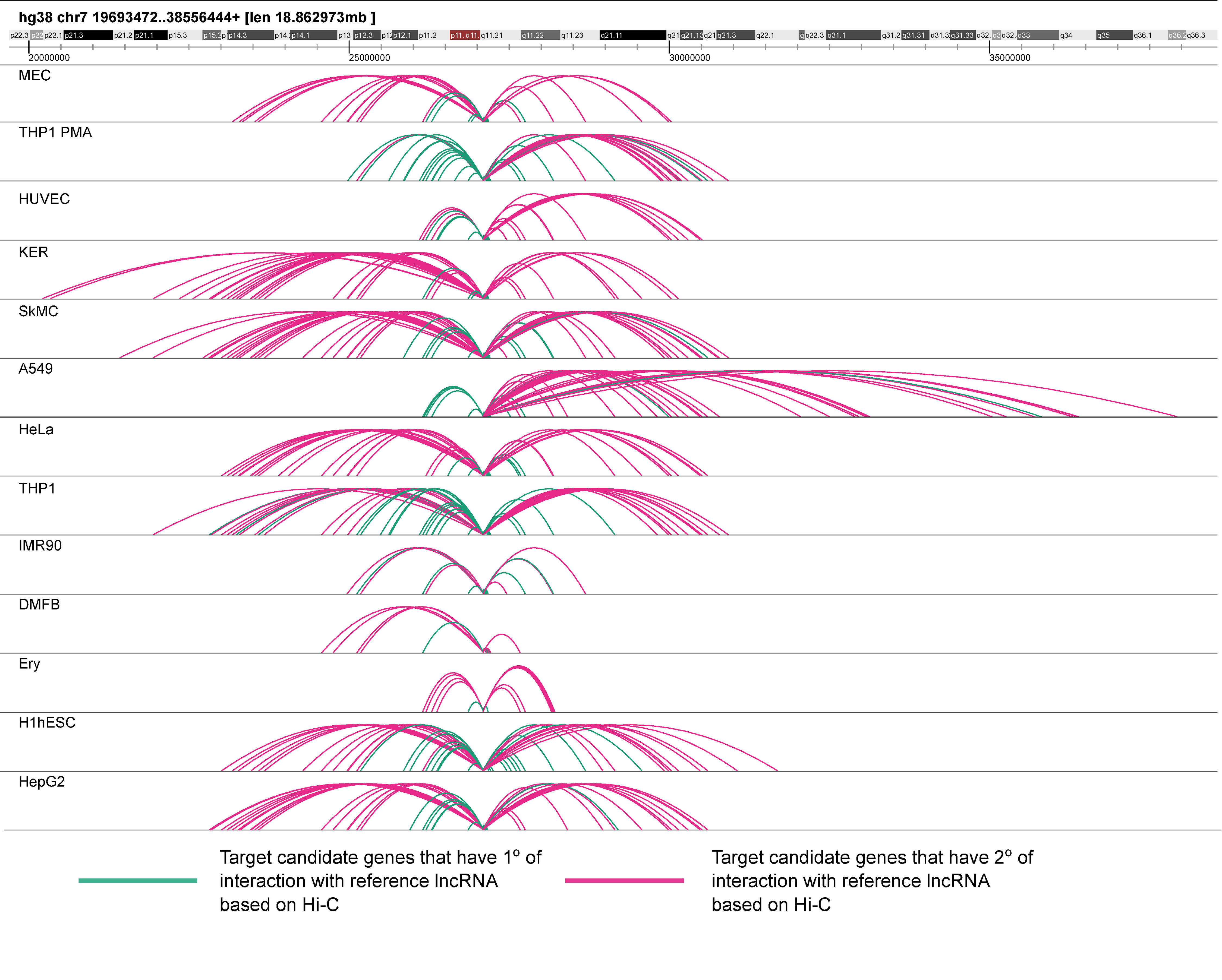

Supplement: S5 Fig — The top track shows the genomic location of the interaction, followed by tracks showing the Hi-C annotated interactions between lncRNA ENSG00000233429 and its candidate target genes in different cell types. (TIF) [file pone.0295971.s005.tif]
